# Sprawling Quadruped Robot Driven by Decentralized Control With Cross-Coupled Sensory Feedback Between Legs and Trunk

**DOI:** 10.3389/fnbot.2020.607455

**Published:** 2021-01-08

**Authors:** Shura Suzuki, Takeshi Kano, Auke J. Ijspeert, Akio Ishiguro

**Affiliations:** ^1^Research Institute of Electrical Communication, Tohoku University, Sendai, Japan; ^2^Japan Society for the Promotion of Science, Tokyo, Japan; ^3^Biorobotics Laboratory, École Polytechnique Fédérale de Lausanne, Lausanne, Switzerland

**Keywords:** sprawling locomotion, body-limb coordination, quadruped robot, decentralized control, sensory feedback control

## Abstract

Quadruped animals achieve agile and highly adaptive locomotion owing to the coordination between their legs and other body parts, such as the trunk, head, and tail, that is, body–limb coordination. This study aims to understand the sensorimotor control underlying body–limb coordination. To this end, we adopted sprawling locomotion in vertebrate animals as a model behavior. This is a quadruped walking gait with lateral body bending used by many amphibians and lizards. Our previous simulation study demonstrated that cross-coupled sensory feedback between the legs and trunk helps to rapidly establish body–limb coordination and improve locomotion performance. This paper presented an experimental validation of the cross-coupled sensory feedback control using a newly developed quadruped robot. The results show similar tendencies to the simulation study. Sensory feedback provides rapid convergence to stable gait, robustness against leg failure, and morphological changes. Our study suggests that sensory feedback potentially plays an essential role in body–limb coordination and provides a robust, sensory-driven control principle for quadruped robots.

## 1. Introduction

Quadrupeds freely locomote in their natural habitat with great agility and efficiency. This agility is achieved by coordination between their legs and other body parts such as the trunk, head, and tail, that is, by body–limb coordination (Hildebrand, 1959; Ashley-Ross, 1994; Reilly and Delancey, 1997; Ijspeert et al., 2007; Jagnandan and Higham, 2017; Ijspeert, 2020). However, most previous studies have intensively investigated interlimb coordination (Aoi et al., 2017), and less attention has been paid to the body–limb coordination mechanisms. A better understanding of these mechanisms can contribute to the design of agile quadruped robots and help to interpret the motor control of quadruped animals.

When investigating body–limb coordination mechanisms, sprawling locomotion can be seen to be a remarkable behavior. A sprawling walking gait combines lateral bending of the body with leg movements. This is exhibited by many amphibians and lizards (Hildebrand, 1959; Ashley-Ross, 1994; Reilly and Delancey, 1997; Ijspeert et al., 2007; Jagnandan and Higham, 2017; Ijspeert, 2020). Lateral bending during locomotion provides a longer stride and stronger thrust, and this behavior was implemented by the first terrestrial quadrupeds (Nyakatura et al., 2019; Ijspeert, 2020). Therefore, this locomotion mode is likely to contain an important characteristic of the body–limb coordination mechanisms.

Sprawling locomotion in vertebrate animals is controlled by a distributed neural network called the central pattern generator (CPG) and sensory feedback from peripheral nerves, according to experiments with salamanders (Cabelguen et al., 2003). Based on these findings, several neural network models have been proposed for sprawling robots to emulate and investigate sprawling locomotion (Ijspeert et al., 2007; Harischandra et al., 2011; Crespi et al., 2013; Yin et al., 2016; Zhong et al., 2018). However, most of the models were based on open-loop control. Thus, the extent to which sensory feedback contributes to shaping body–limb coordination was not investigated, particularly in studies using real robots. Clarifying the role of sensory feedback in sprawling locomotion is an important step in understanding the fundamental principles of body–limb coordination.

Our motivation is to understand the role of sensory feedback in sprawling locomotion. Our previous study proposed decentralized control with cross-coupled sensory feedback, that is, bidirectional feedback from body to limb and limb to body in a simulated robot (Suzuki et al., 2019). The simulated results showed that sensory feedback helps to rapidly establish appropriate body–limb coordination. Moreover, sensory feedback provides adaptability to leg failure and changes in the body aspect ratio. This paper presents the experimental validation of the proposed control using a developed quadruped robot. The robot was designed based on the simulated robot in the previous study, and it was equipped with servo motors with built-in torque sensors. The results show a tendency similar to that of the simulation. This suggests that cross-coupled sensory feedback potentially plays an essential role in body–limb coordination. It could be a useful concept for designing decentralized and robust controllers for quadruped robots.

The remainder of this paper is structured as follows. Section 2 describes the developed quadruped robot and decentralized control with cross-coupled sensory feedback. Section 3 describes the experimental setup, results, and discussion. In section 4, the conclusions and recommendations for future studies are presented.

## 2. Robot and Control Algorithm

### 2.1. Mechanical System

**Figure 1:**
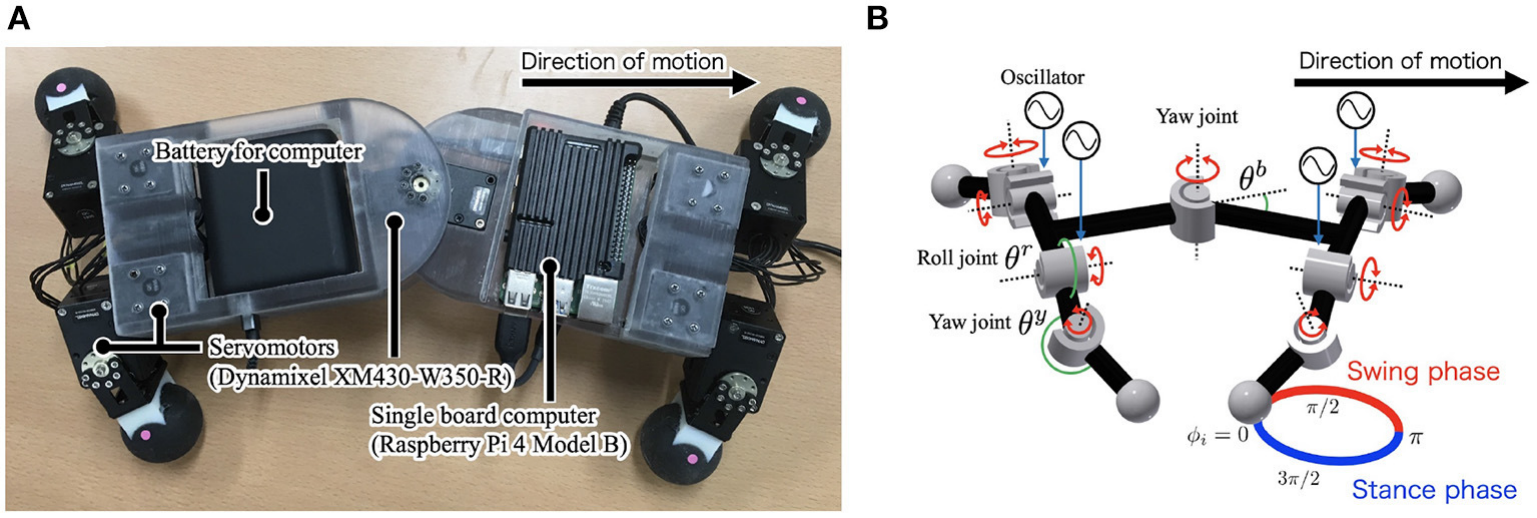
**(A)** Twister, a quadruped robot that exploits lateral bending. Body height 0.09 m, width 0.095 m, length 0.30 m, leg length 0.07 m, and weight 2.1 kg. **(B)** Schematic of the robot. The trunk has one servomotor, and each leg has two servomotors controlled by the phase of the oscillators ϕ_*i*_. θ^*b*^ is the trunk angle, and θ^*y*^ and θ^*r*^ are the angles of the leg actuators in the yaw and roll directions, respectively.

Figure 1A shows the developed quadruped robot *Twister* with nine actuated degrees of freedom (DoFs): one actuated DoF in the trunk and two actuated DoFs per leg. It is primarily constructed using 3D-printed ABS (acrylonitrile-butadiene-styrene) resin pieces. The actuated DoFs were realized with servomotors (Dynamixel XM430-W350-R, ROBOTIS, stall torque 4.1 [N·m] at 12 V) that were controlled by a single-board computer (Raspberry Pi 4 Model B, 4 GB RAM, OKdo). The robot consists of a trunk segment and four leg segments. The trunk segment has a rotary actuator in the yaw direction. Each leg segment has two rotary actuators in the yaw and roll directions, along with a phase oscillator that controls the leg segment.

Current and angle sensors are included in each servomotor. The current values are proportional to the motor torque, so the current sensor is used as a torque sensor. An angle sensor at the trunk detects the trunk-joint angle θ^*b*^. θ^*b*^ is positive when the trunk-joint bends to the right, as shown in Figure 1B.

### 2.2. Control Algorithm

**Figure 2:**
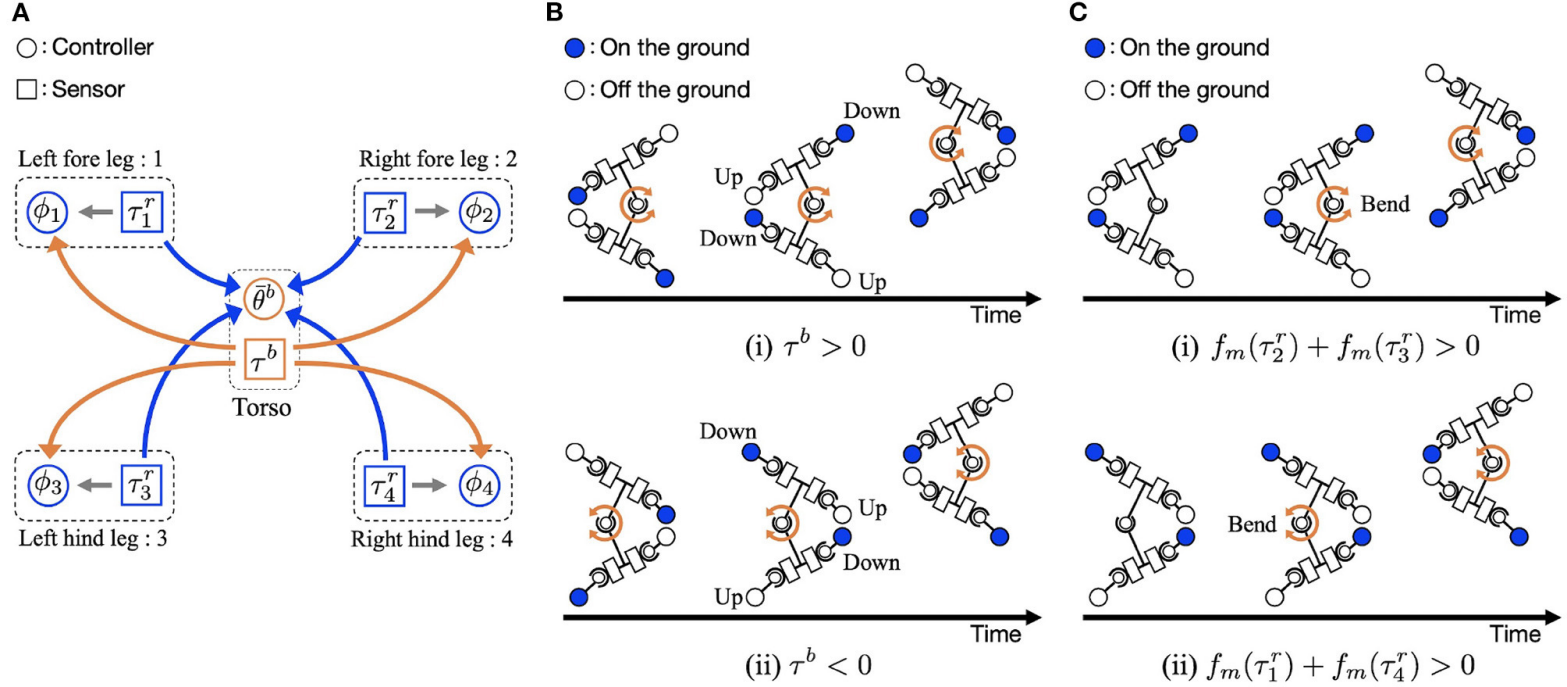
Overview of the control algorithm. **(A)** Feedback structure: Control variables ϕ_*i*_ and ${\bar{\theta }}^{b}$ are determined by three types of sensory feedback using the torque values at the leg actuator in the roll direction $\tau_{i}^{r}$ and the trunk τ^*b*^ as sensory information. Each leg number is designated by suffix *i*. **(B)** Body-to-limb sensory feedback mechanism: (i) The trunk actuator bends the body to the right (τ^*b*^ > 0). (ii) The trunk actuator bends the body to the left (τ^*b*^ < 0). **(C)** Limb-to-body sensory feedback mechanism: (i) right fore and/or left hind limbs are on ground [$f_{m}\left(\tau_{2}^{r}\right)+f_{m}\left(\tau_{3}^{r}\right)>0$], and (ii) left fore and/or right hind limbs are on the ground [$f_{m}\left(\tau_{1}^{r}\right)+f_{m}\left(\tau_{4}^{r}\right)>0$].

This section describes decentralized control with cross-coupled sensory feedback slightly modified from the control algorithm in our previous study (Suzuki et al., 2019), for robot control. The controller is made from oscillators, which represent CPGs. Unlike most CPG controllers, the controller does not use inter-oscillator couplings but sensory-couplings through bidirectional feedback between the legs and the trunk (Figure 2A). The sensory couplings were achieved using the following three feedback rules:

1. Torque sensory feedback from limb to limb
2. Torque sensory feedback from body to limb
3. Torque sensory feedback from limb to body

The first rule is responsible for coordinating the four legs as they move forward while supporting the body. The second and third rules comprise bidirectional feedback that establishes self-organized body–limb coordination. The controller generates stable and flexible sprawling locomotion by the combination of oscillators generating rhythmic motion and the feedback rules coordinating the movements of bodily DoFs. While the controller is programmed here on a single computer, it is ideally suited for a distributed implementation on different independent microcontrollers, for example, one for the trunk and one per leg, with minimal communication between microcontrollers sharing sensory signals. The following section describes the control algorithm and the effects of sensory feedback.

#### 2.2.1. Leg Control

A phase oscillator is implemented in each leg, and its phase determines the target angle of the rotary actuators in the yaw and roll directions as follows:

(1) $$\begin{array}{l} {\bar{\theta }}_{i}^{y}=C_{0}^{y}-C_{amp}^{y}\cos\phi_{i},\\ {\bar{\theta }}_{i}^{r}=C_{0}^{r}-C_{amp}^{r}\sin\phi_{i}, \end{array}$$

where ${\bar{\theta }}_{i}^{y}$ and ${\bar{\theta }}_{i}^{r}$ denote the target angles, $C_{0}^{y}$ and $C_{0}^{r}$ represent the neutral angles, $C_{amp}^{y}$ and $C_{amp}^{r}$ represent the amplitude of the yaw and roll actuators, respectively (Figure 1B). ϕ_*i*_ is the oscillator phase. When 0 < ϕ_*i*_ < π, the leg is in the swing phase; otherwise, it is in the stance phase. The suffix *i* denotes the leg identifier (1: left fore, 2: right fore, 3: left hind, and 4: right hind). The time evolution of the phase is described as follows:

(2) $$\begin{array}{l} {\dot{\phi }}_{i}=\omega +f_{LL,i}+f_{BL,i}, \end{array}$$

(3) $$\begin{array}{l} f_{LL,i}=-\sigma_{LL}f_{m}\left(\tau_{i}^{r}\right), \end{array}$$

(4) $$\begin{array}{l} f_{BL,i}=\{\begin{array}{ll} \sigma_{BL}\tau^{b}\cos\phi_{i} & \left(i=1,4\right)\\ -\sigma_{BL}\tau^{b}\cos\phi_{i} & \left(i=2,3\right), \end{array} \end{array}$$

(5) $$\begin{array}{l} f_{m}\left(\tau_{i}^{r}\right)=\max\left[0,\tau_{i}^{r}-\tau_{th}^{r}\right], \end{array}$$

where ω [rad/s] denotes the intrinsic angular velocity of the phase oscillators, and σ_*LL*_ [rad/N·m·s] and σ_*BL*_ [rad/N·m·s] are the weights of the sensory feedback terms. $\tau_{i}^{r}$ [N·m] and τ^*b*^ [N·m] represent the torques at the leg actuator in the roll direction and at the trunk actuator, respectively. $f_{m}\left(\tau_{i}^{r}\right)$ is correlated with the extent to which the leg supports the body. Thus, $f_{m}\left(\tau_{i}^{r}\right)$ substitutes for the ground reaction force (GRF) information. $\tau_{th}^{r}$[N·m] is a constant positive value as the threshold of the sensors.

Equation (3) works according to the feedback from limb to limb. The local feedback rule was proposed by Owaki et al. (2013). It generates adaptive interlimb coordination in response to the speed and physical properties of the robot (Owaki et al., 2013; Owaki and Ishiguro, 2017). Based on the sensory feedback effect, the oscillator phase is modulated to 3π/2 when $f_{m}\left(\tau_{i}^{r}\right)>0$. When the leg supports the body, the roll joint of that limb has higher torque signals, that is, higher $f_{m}\left(\tau_{i}^{r}\right)$. Thus, this feedback means that the leg remains on the ground when it supports the body. The local sensory information $f_{m}\left(\tau_{i}^{r}\right)$ describes the extent to which a specific leg provides support to the body, and also indicates how much other legs are currently contributing to supporting the body. Using the sensory information, this feedback can generate adaptive interlimb coordination without neural communication between the legs.

Equation (4) relates to the feedback from the body to the limb (Figure 2B). When the trunk actuator bends the body to the right (τ^*b*^ > 0), the oscillator phases of the left fore and right hind legs are modulated toward π/2 to lift the legs, and the oscillator phases of the right fore and left hind legs are modulated toward 3π/2 to place the legs on the ground. By phase modification, the left fore and right hind legs lift from the ground, and the other legs are anchored on the ground. This facilitates the trunk actuator bending the body to the right (θ^*b*^ > 0), and the robot moves forward when the anchored legs serve as a pivot.

#### 2.2.2. Body Control

The time evolution of the target angle of the trunk actuator is described as follows:

(6) $$\begin{array}{l} {\dot{\bar{\theta }}}^{b}=a\left(-\theta^{b}+f_{LB,i}\right), \end{array}$$

(7) $$\begin{array}{l} f_{LB,i}=\sigma_{LB}\tanh\left\{\rho \left(-f_{m}\left(\tau_{1}^{r}\right)+f_{m}\left(\tau_{2}^{r}\right)+f_{m}\left(\tau_{3}^{r}\right)-f_{m}\left(\tau_{4}^{r}\right)\right)\right\}, \end{array}$$

where *a* [1/s] represents the reciprocal of the time constant. Variables θ^*b*^ and ${\bar{\theta }}^{b}$ are the actual angle and target angle of the trunk actuator, respectively. σ_*LB*_ [rad] and ρ [1/N·m] represent the weights of the sensory feedback.

Equation (7) relates to the feedback from limb to body. The sensory feedback effect is that the trunk bends in response to ground contact, as shown in Figure 2C. When the right fore and/or left hind limbs are on the ground [$f_{m}\left(\tau_{2}^{r}\right)+f_{m}\left(\tau_{3}^{r}\right)>0$], the actuator makes the right side of the body concave (τ^*b*^ > 0, Figure 2Ci). Similarly, when the left fore and/or right hind limbs are on the ground [$f_{m}\left(\tau_{1}^{r}\right)+f_{m}\left(\tau_{4}^{r}\right)>0$], the trunk actuator makes the left side of the body concave (τ^*b*^ < 0, Figure 2Cii). The interaction of the sensory feedback from body to limb and limb to body establishes the relationship between the legs and trunk, providing longer strides and more powerful pushing off against the ground.

## 3. Robot Experiments

To verify the control algorithm in the real world, we conducted three experiments: (i) steady locomotion, (ii) fault tolerance, and (iii) robustness to change in body aspect ratio. All experiments were conducted on flat terrain and recorded with a video camera (Cyber-shot DSC-RX0M2, Sony). The video of representative results can be referred to in Supplementary Material. The parameter values were as follows: $C_{0}^{y}=\pi$ [rad], $C_{0}^{r}=7\pi /6$ [rad], $C_{amp}^{y}=\pi /12$ [rad], $C_{amp}^{r}=\pi /12$ [rad], ω = 1.5π [rad/s], σ_*LL*_ = 6.83 [rad/N·m·s], σ_*BL*_ = 2.28 [rad/N·m·s], σ_*LB*_ = π/6 [rad], ρ = 0.05 [1/N·m], $\tau_{th}^{r}=0.088$ [N·m], *a* = 5.0 [1/s]. Here, most of the parameter values were set to be identical to those in our simulation study (Suzuki et al., 2019). However, several parameters were adjusted from these values by trial-and-error because of differences in the mechanical and morphological properties between the real and simulated robots. The analysis was conducted using MathWorks' MATLAB for gait classification and Tracker, a free video analysis and modeling tool, for speed derivation.

### 3.1. Steady Locomotion

**Figure 3:**
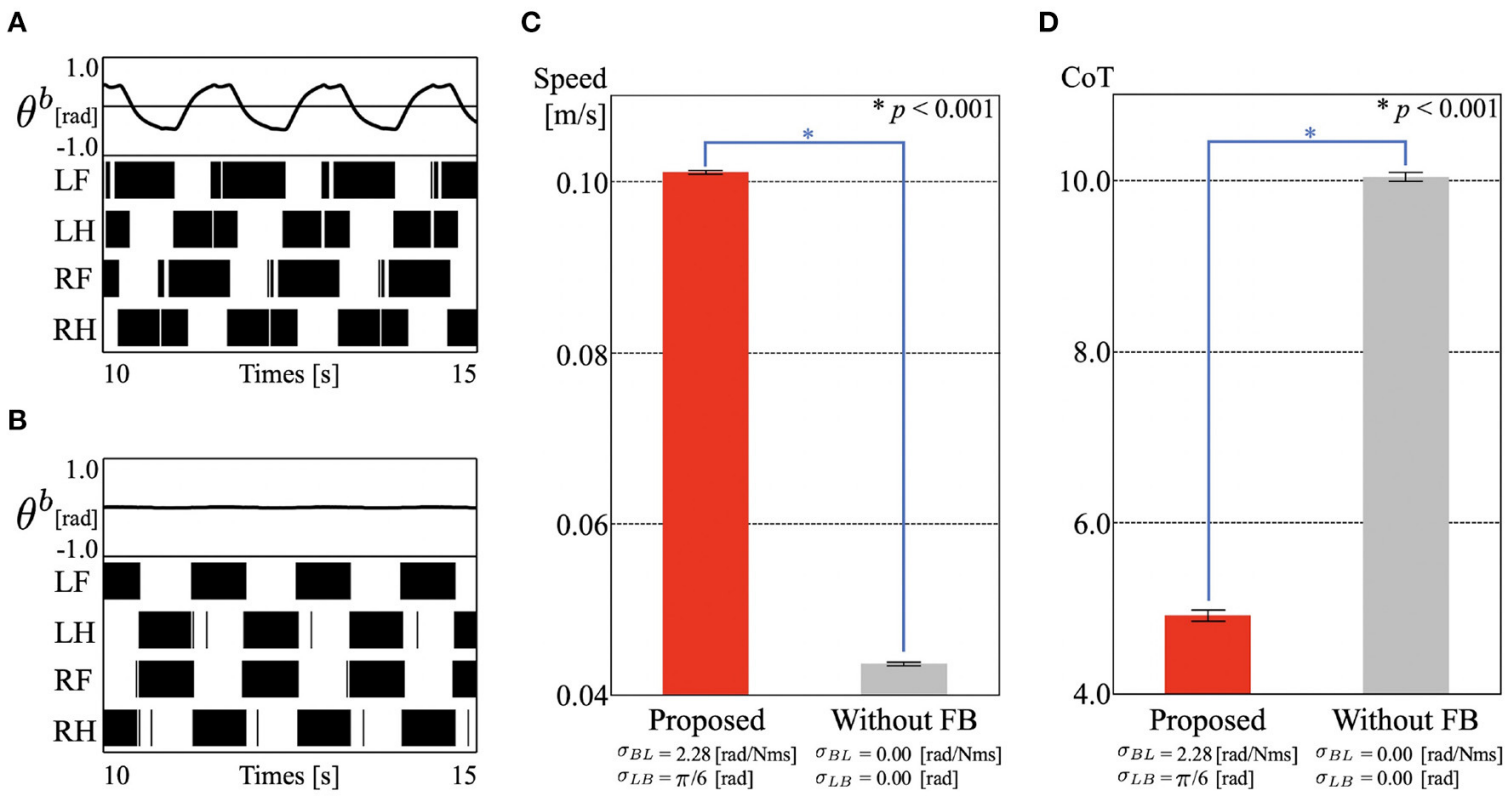
Experimental results of section 3.1. **(A)** Gait pattern of proposed control. Time evolution of (top) trunk-joint angle θ^*b*^ and (bottom) gait diagrams. The colored region in the gait diagrams represents the stance phase of each leg [$f_{m}\left(\tau_{i}^{r}\right)>0$]. **(B)** Gait pattern of the control without cross-coupled sensory feedback [σ_*LB*_ = σ_*BL*_ = 0]. **(C)** Locomotion speed of the proposed control (0.1010 [m/s], SD = 0.0002), and without cross-coupled sensory feedback (0.0436 m/s, SD = 0.0002). The bar heights and the error bars indicate means and standard deviations (SD) for five trials, respectively (^*^p < 0.001, *t*-test). **(D)** Cost of transport (CoT) of the proposed control (CoT = 4.91, SD = 0.0646), and without cross-coupled sensory feedback (CoT = 10.04, SD = 0.0487).

First, we observed the manner of locomotion from two points of view: footfall patterns and the angle of the trunk-joint. The results are shown in Figure 3A and Supplementary Video 1. Figure 3A show the time evolution of (top) the trunk-joint angle θ^*b*^ and (bottom) the gait diagrams, where the colored region represents the stance phases [$f_{m}\left(\tau_{i}^{r}\right)>0$]. The duty factor is 61.0%, and the diagonality is 42.2%. The duty factor is the time percentage that one foot spends in the stance phase during a gait cycle, and diagonality is the percentage of the cycle period by which the left/right hind footfall precedes the left/right fore footfall. Thus, the resulting gait is classified as a lateral-sequence walking gait, in which the feet touch down in the order right hind (RH), right fore (RF), left hind (LH), and left fore (LF), in Hildebrand's gait classification (Hildebrand, 1965; Cartmill et al., 2002). It is qualitatively similar to the sprawling locomotion shown in animals (Ashley-Ross, 1994; Reilly and Delancey, 1997). In addition, the robot converged to this gait in a few steps, even though we set the initial phase of all oscillators to be the same (ϕ_1_ = ϕ_2_ = ϕ_3_ = ϕ_4_ = 3π/2, Supplementary Video 1). This is due to the effect of the feedback. (Owaki et al., 2013) showed that the feedback from limb to limb (Equation 3) provides smoothly interlimb coordination. Additionally, in our model, the feedback from body to limb and from limb to body (Equation 4, Figure 2B) helps establish stable locomotion with body–limb coordination. This means that the robot produces the appropriate relationship between the legs and trunk spontaneously and immediately without a predefined body–limb relationship, as in conventional studies. This was the same as in the simulation results (Suzuki et al., 2019).

Next, we investigated the contribution of the cross-coupled sensory feedback to locomotion performance. We derived the locomotion speed and CoT from the tracking data and compared them for the proposed control with and without cross-coupled sensory feedback between the legs and trunk (σ_*LB*_ = σ_*BL*_ = 0). Here, steady locomotion without cross-coupled sensory feedback is shown in Figure 3B and Supplementary Video 2. Stable walking was also exhibited in a few steps, because the controller still includes the limb to limb sensory feedback. The gait is classified as a walking trot gait, the duty factor is 53.2%, and the diagonality is 48.5%. The CoT is calculated as follows:

(8) $$\begin{array}{l} \text{CoT}=\frac{1}{Dmg}\int_{0}^{T}\sum P_{i}\left(t\right)\text{d}t, \end{array}$$

where *D* [m] is the travel distance, *m* [kg] is the total mass of the robot, *g* [m/s^2^] is the gravitational acceleration, and *P*_*i*_(*t*) [W] is the power consumption of each servomotor. Figures 3C,D show the mean speed and CoT for each control mechanism, where the height of the plots and the error bars represent the mean values and standard deviations (SD) for five trials, respectively. We used Welch's *t*-test as a statistical test to compare the controllers. From the perspectives of locomotion speed and CoT, the proposed control mechanism achieved significantly higher speeds and energy efficiency than those of the control mechanism without cross-coupled sensory feedback (*p* < 0.001). The results show that lateral bending of the body with leg movements improves mobility, and that cross-coupled sensory feedback contributes to this.

### 3.2. Fault Tolerance

In this experiment, we investigated the robustness to leg failure. Here, leg failure means that the leg becomes stuck in a particular position. Specifically, the phase of the leg oscillator is fixed to 3π/2, and therefore, the target angle of the leg actuator is also fixed to ${\bar{\theta }}_{i}^{y}=C_{0}^{y}$, ${\bar{\theta }}_{i}^{r}=C_{0}^{r}+C_{amp}^{r}$, respectively. We derived the locomotion speed and CoT for the proposed control mechanism under the foreleg (ϕ_1_ = ϕ_2_ = 3π/2) and hindleg failure conditions, (ϕ_3_ = ϕ_4_ = 3π/2). These were compared with those of the open-loop trot control mechanism to consider the importance of sensory feedback in an unexpected situation.

The open-loop trot control mechanism predefined the relationship between the legs and the trunk without any sensory feedback. Each actuated DoF has a phase oscillator that defines the target angle as follows:

(9) $$\begin{array}{l} {\dot{\phi }}_{i}=\omega^{\prime }, \end{array}$$

(10) $$\begin{array}{l} \phi_{b}=\phi_{1}, \end{array}$$

(11) $$\begin{array}{l} {\bar{\theta }}^{b}=C_{amp}^{b}\sin\phi_{b}, \end{array}$$

where ω′ [rad/s] denotes the intrinsic angular velocity of the phase oscillators. ϕ_*b*_ [rad] is the phase of the oscillator in the trunk, and $C_{amp}^{b}$ represents the amplitude of the trunk actuator. The parameters ω′ and $C_{amp}^{b}$ were defined to refer to the gait cycle and the trunk-joint angle of the proposed model (Figure 3A), as follows: ω = 4.363 [rad/s] and $C_{amp}^{b}=0.43$ [rad]. The initial conditions of the oscillator are ϕ_1_ = ϕ_4_ = 0, ϕ_2_ = ϕ_3_ = π. Steady locomotion with the open-loop control mechanism is shown in Supplementary Video 3.

**Figure 4:**
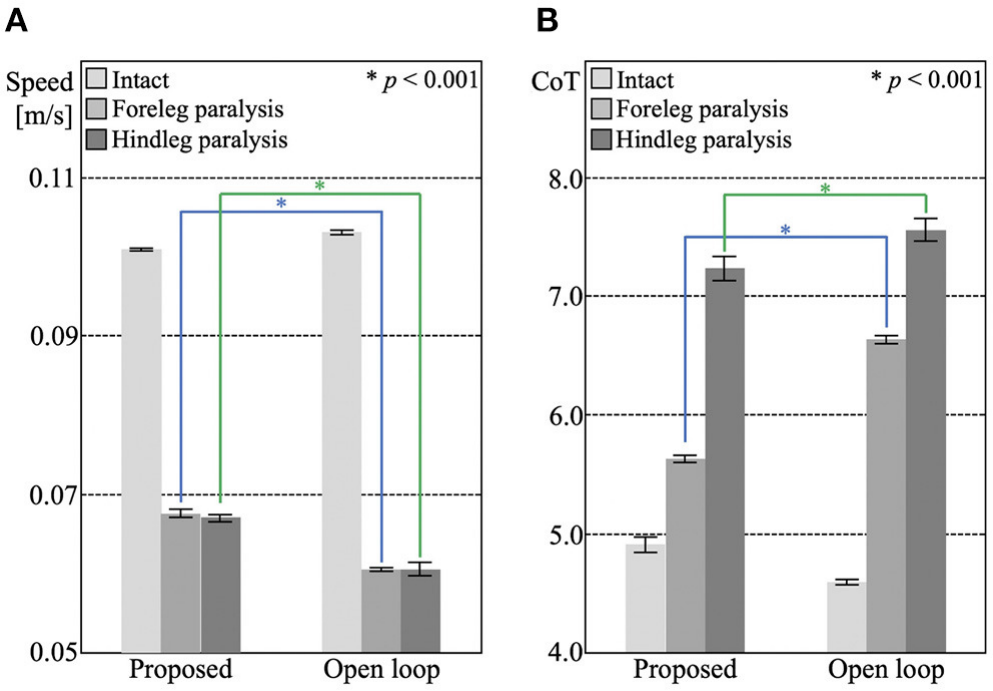
The experimental results of section 3.2. The bar heights and the error bars indicate the means and SD for five trials, respectively (^*^p < 0.001, *t*-test). **(A)** Locomotion speed of the proposed control under the intact condition (0.1010 [m/s], SD = 0.0002), foreleg failure condition (0.0675 [m/s], SD = 0.0005), and hindleg failure condition (0.0670 [m/s], SD = 0.0004), and of open-loop trot control under the intact condition (0.1031 m/s, SD = 0.0003), foreleg failure condition (0.0605 m/s, SD = 0.0002), and hindleg failure condition (0.0605 [m/s], SD = 0.0008). **(B)** CoT of the proposed control under the intact condition (CoT = 4.91, SD = 0.0646), foreleg failure condition (CoT = 5.63, SD = 0.031), and hindleg failure condition (CoT = 7.24, SD = 0.102), and of open-loop trot control under intact condition (CoT = 4.59, SD = 0.0211), foreleg failure condition (CoT = 6.63, SD = 0.036), and hindleg failure condition (CoT = 7.56, SD = 0.095).

The locomotion during each leg failure condition is shown in Supplementary Videos 4–7. Figure 4 shows the locomotion speed and CoT of the proposed and open-loop trot control under intact, foreleg failure, and hindleg failure conditions. The proposed control showed significantly higher speeds and energy efficiency than the open-loop trot control under the leg failure conditions (*p* < 0.001), although the opposite tendency was observed in the intact condition. This result suggests that the sensory feedback mechanism works well to adapt to unexpected bodily damage, although it does not yield the same performance as the open-loop control when intact.

### 3.3. Robustness to Changes in Body Aspect Ratio

We observed locomotion when the leg length was elongated (length = 0.14 [m]) to investigate the robustness to variations in the body aspect ratio. The observed locomotion is shown in Supplementary Video 8. The mean speed was 1.735 [m/s], and CoT was 5.25 for five trials. The robot with long legs showed stable locomotion similar to the one with short legs (length = 0.07 [m], Supplementary Video 1). Moreover, it did not require any changes in the control parameters for the morphological changes. This highlights the robustness of the control algorithm against variations in the body aspect ratios.

## 4. Conclusion and Future Work

We have developed a quadruped robot and demonstrated via experiments that decentralized control with cross-coupled sensory feedback (Suzuki et al., 2019) enables effective sprawling locomotion. Unlike most previous works based on CPGs with inter-oscillator couplings (Ijspeert et al., 2007; Crespi et al., 2013; Yin et al., 2016; Ijspeert, 2020), or on gait patterns based on geometric mechanics (Zhong et al., 2018), our model uses sensory-couplings through bidirectional feedback between the legs and trunk (Figure 2A). Owing to this mechanism, the robot can quickly converge to stable gaits, achieve high locomotion performances, and adapt to leg failure. Interestingly, these gaits are obtained through an emergent property of controller-body-environment interactions unlike the fixed gait patterns of previous work. And the generated gaits are highly similar to those found to be optimal in terms of forward speed through geometric mechanics (Zhong et al., 2018). Our results suggest that the sensory feedback mechanisms at the peripheral level play an important role in coordinating the body and limbs.

This work will also provide insight into motor control underlying other legged locomotion. Many modeling studies of six-legged walking have investigated the importance of sensory feedback (Manoonpong et al., 2013; Schilling et al., 2013; Dürr et al., 2019; Schilling and Cruse, 2020). In particular, Schilling et al. (2013) proposed a decentralized mechanism similar to that used in this study. This indicates the similarities of motor control between six-legged and four-legged locomotion. Through examining the importance of body motion in legged locomotion, Schilling et al. (2012) indicated the significance of trunk-joints in six-legged walking and turning, and Ly et al. (2011) showed that the bio-inspired vertebral column enhances humanoid balance. Furthermore, Fukuhara et al. and Kano et al. used similar feedback mechanisms, and found that the body bending along the pitching direction increases locomotion speeds of a cheetah-like robot (Fukuhara et al., 2020) and sea roach robot (Kano et al., 2019), respectively. Our results also suggest the importance of sensory feedback and body–limb coordination in legged locomotion. This indicates that further study of cross-coupled sensory feedback would contribute to understanding legged locomotion not limited to sprawling locomotion.

This work also contributes to the field of robotics. Most previous studies on legged robots mainly focused on leg motion rather than whole-body motion. In contrast, this study achieved effective locomotion with lateral bending using a simple control framework. Furthermore, because the framework was designed for decentralized implementation, it can provide fault tolerance and robustness with low computational cost and local sensing. Therefore, this work is expected to provide fundamental information for the next paradigm of fault-tolerant legged robots.

Finally, we point out several problems and limitations with this work and potential solutions for them. First, a neurophysiological basis for the proposed model is still lacking. Although it is known that the neural circuit for limb movements is located in the particular vertebrae above and below the axial trunk network (Bicanski et al., 2013; Ryczko et al., 2020), it is still unclear whether salamanders share proprioceptive information between the legs and trunk for locomotion, as in the proposed controller. This needs to be further investigated. Second, the parameters have not been explored sufficiently as compared with our simulation study (Suzuki et al., 2019). As a consequence, it is still unclear to what extent the behavior is sensitive to parameter changes. Clarifying this will help identify the crucial parameters for locomotion. Third, the experiments were limited to straight walking, and turning behaviors were not investigated. We expect that the turning direction can be controlled by modulating the feedback from limbs to body in an asymmetric way (for example, the robot would turn right by removing the sensory information for the left foreleg). Fourth, the robot developed in this study has only one DoF in the trunk, whereas salamanders have many DoFs. In the future, we would like to implement control in a robot with a multi-DoF trunk and compare its behavior with that of real salamanders.

## Data Availability Statement

The original contributions presented in the study are included in the article/Supplementary Material, further inquiries can be directed to the corresponding author/s.

## Author Contributions

SS, AJI, and AI designed the study. AI supervised the project. SS developed the robot platform. SS, TK, and AI designed the control algorithm. SS carried out the robot experiments and the initial draft of the manuscript. All authors revised the manuscript.

## Conflict of Interest

The authors declare that the research was conducted in the absence of any commercial or financial relationships that could be construed as a potential conflict of interest.
